# Child’s assent in research: Age threshold or personalisation?

**DOI:** 10.1186/1472-6939-15-44

**Published:** 2014-06-13

**Authors:** Marcin Waligora, Vilius Dranseika, Jan Piasecki

**Affiliations:** 1 Department of Philosophy and Bioethics, Faculty of Health Sciences, Jagiellonian University, Medical College, Michalowskiego 12, Krakow 31-126, Poland; 2 Department of Logic and History of Philosophy, Vilnius University, Vilnius, Lithuania

**Keywords:** Children, Research, Paediatrics, Assent, Vulnerable subjects

## Abstract

**Background:**

Assent is an important ethical and legal requirement of paediatric research. Unfortunately, there are significant differences between the guidelines on the details of assent.

**Discussion:**

What often remains unclear is the scope of the assent, the procedure for acquiring it, and the way in which children’s capacity to assent is determined. There is a general growing tendency that suggests that the process of assent should be personalised, that is, tailored to a particular child. This article supports the idea of personalisation. However, we also propose placing limits on personalisation by introducing a suggested requirement of assent starting at a school-age threshold. In some situations RECs/IRBs and researchers could reduce the suggested threshold.

**Summary:**

A recommended age threshold is likely to serve the interests of children better than ambiguous and flexible criteria for personalised age determination.

## Background

One of the most important ethical and legal principles that results from respect for the autonomy of participants in biomedical research is the voluntary nature of participation. In order to secure this, many countries have adopted a policy of providing research participants with basic written information together with a suggestion for researchers to discuss any extra details that the potential participant may wish to learn more about [1]. For people taking part in research who do not have full capacity, informed consent must be acquired from a legal guardian or a representative. Often the assent of a research participant without full capacity is also needed. Most international guidelines on the ethics of human research stress that the principle of assent must also be applied in paediatric research [2-6]. However, sometimes the concept of assent is criticised as a contentious term which, by replacing children’s consent, weakens their position in the decision-making process [7]. Moreover, what often remains unclear is the scope of this assent, the procedure for acquiring it, and the way in which children’s capacity to assent is determined. For nearly 30 years, commentators have been discussing a number of different aspects of assent and its personalisation. For example, Priscilla Alderson and Jonathan Montgomery recommended that all discussions with children regarding participation in research should start from the presumption of children’s competence, rather than the assumption that the child is incompetent [8]. Phaik Yeong Cheah and Michael Parker argue that, especially in some low-income settings, children may be better informed and educated than their parents, and thus in some cases should be able not to assent but to consent for themselves [9]. Other authors are in favour of an increase in the role of parents in taking decisions for the children to participate in the research. According to Tessa John et al., parents should interpret their child’s behaviour and statements, assessing the significance of their dissent and assent [10]. Barbara E. Gibson et al. propose implementing a “family decision-making” model [11]. They argue that joint family consent should replace separate parental consent and child’s assent. A similar opinion is held by Steven Joffe, who at the same time recommends rejecting the term “assent” as not entirely clear, in favour of “affirmative agreement” [12]. Paul Baines proposes abandoning the requirement to obtain assent entirely. If children are incompetent, he argues, we should not ask them for assent. Yet if they are competent, they should be able to express not assent, but consent [13]. Noor A. A. Giesbertz et al. argue the case for the introduction of personalised assent, saying that the value of assent is not in assuring the safety of participation, but in engagement grounds. Assent helps to develop a child’s autonomy, plays the role of educational tool, and supports communication between the researcher and the child. The content and the process of decision making in assent should thus be adjusted to the individual child [14-16]. Other authors claim that, in the process of the parental and child’s decision making, advanced methods could help with transmitting knowledge of the research, making it easy to understand for the parents as well as the children [17].

We believe that assent is an important part of the current paradigm of free and voluntary participation in biomedical research, and also support the view that assent plays an important role in the process of the development of a child’s autonomy. In general, we are sympathetic to many aspects of the idea of personalised assent. In this article, however, we will focus solely on the problem of personalised determination of age of assent. Our proposal is that personalisation should be limited by a suggested requirement of assent starting at a certain age threshold.

## Discussion

### Personalisation of the age of assent

According to some of the international instruments regulating human research, the power of influence of a child’s assent should increase with age [4,5]. Some professional and national recommendations, meanwhile, specify an unambiguous age limit for children that marks the need for acquisition of assent. The UK Royal College of Paediatrics defines this as “school age” [18]. In the United States, the National Commission for the Protection of Human Subjects of Biomedical and Behavioral Research sets this point at a child’s seventh birthday [19]. The American Academy of Pediatrics also supported this age threshold until 2010 [20], but now applies less strict requirements [21].

There is a general growing tendency in favour of personalisation of the process of assessment of the age of assent, i.e. suggesting that it should be tailored to a particular child. Here commentators use different arguments. Firstly, large developmental differences between children of the same age and differences in experience influence a child’s ability to understand essential information, such as the purpose, risk and benefit of research. In particular, children undergoing long-term medical treatment demonstrate surprising competences concerning their own medical situation [22]. Secondly, especially with smaller children, their verbal and non-verbal messages demand advanced interpretation and assessment, thus making it difficult for a research team to assess the maturity and level of understanding of a child. Most authors agree that in this case an important role should be played by the parents, who are the best at interpreting the behaviours of their own children. Parents could also be asked whether the child is old and competent enough to assent [10,11,23].

Unfortunately, the whole interesting idea of personalisation of age of assent makes the entire process difficult to enforce: it implies sophisticated and time-consuming methods of interpretation of children’s behaviours without clear instruments of assessment and, if not made obligatory, is likely to be avoided when possible. In addition, a joint decision taken by the family and child assumes a certain model of family relations. This model is characterised by the mutual understanding of the family members, parental protectiveness, care and attention in supporting the children’s aspirations. The researchers described in this model are characterised by high moral integrity. Personalised assent falls short entirely in perhaps rare cases when parents do not have the necessary skills for communicating properly with their children. As a result, parents do not know accurately enough what the children’s needs and preferences are. In some cases parents may also simply attach less importance to acting for their children’s good, and as a consequence, may not act in their child’s best interest. In such circumstances it is likely that the family will assign little significance to the assent of the child. It will be a similar story when the researcher is above all interested in the speed of recruitment. With some clinical trials, assent must be acquired within a matter of days or even hours, and there is no time for subtle interpretations and recontact [24]. A comprehensive proposal regarding acquisition of assent cannot exclude the abovementioned situations and institutional problems, and needs further discussion. Otherwise, there is a real risk that extensive, sometimes unclear and incompatible ethical recommendations would be applied very inconsistently. This is true of the Polish part of the large multicentre GABRIEL study, where assent was not sought.

### A case from the Gabriel project

The GABRIEL project was the largest study to date on the genetics of asthma [25]. The research included 10,365 people diagnosed with asthma and 16,110 unaffected people. Part of the GABRIEL project study took place in Poland. This section of the study was conducted in schools located in rural areas and small towns. In the first phase of the study, questionnaires on asthmatic complaints were collected from parents of affected children, who were sent a letter with information on the study and a consent form regarding the realisation of the second phase. Almost a quarter of parents (9,677 out of a total of 40,000) gave their consent to their child’s participation in the second phase of the study. This comprised both obtaining further information from the parents and a qualified nurse taking a blood sample and nasal swab and performing a prick skin test on school grounds. These tests were conducted on a random selection of children from the group of 9,677 [26,27].

After the second phase, a further visit took place in schools, during which additional information was acquired, including asking the children for their opinions on participation in the main genetic study [27]. Distribution of the “participation questionnaire” began at the point when around half of these second school visits were complete. A total of 706 children aged 6–14 filled in the additional form. This supplementary study gives us detailed information on the participation of children in the decision-making process in relation to their participation in the genetic research.

The results of the study by Sylvia van der Pal et al. are rather surprising. The study protocol on genetic research did not foresee any form of joint decision by children and parents on whether they would take part in the genetic research: “The children were informed about the study by the research team but were not officially asked for their assent as part of the official study protocol” [27]. According to Polish law it is possible to obtain written consent for participation in biomedical research of minors aged 16 and older. It is also necessary, in addition to the consent of a legal representative, to acquire written consent from minors under 16, provided that they are able to give an informed opinion regarding participation: “The participation of juveniles in medical experimentation is permitted only provided that written informed consent of a legal representative is obtained. When a juvenile is aged 16 years or younger and is able to give a duly informed opinion about his/her participation in the experiment, his/her written consent is also required.” [28].

Van der Pal et al.’s article lacks any information as to whether any criteria of capability of children were applied, or whether it was decided that even the oldest of the children participating in the research did not possess the capability to give an informed opinion on their participation.

Although Polish law does in fact not contain such a requirement, obtaining assent and determining dissent constitute a standard according to many international guidelines. However, most recommendations are vague, and leave the evaluation of a child’s capability up to the researcher, Regional Ethics Committees (RECs), or Institutional Review Boards (IRBs). There is also a lack of clear procedures determining children’s capability. Some commentators are in favour of an increase in the influence of parents on the procedure of assent or replacing the separate consent of parents and assent of children with a joint decision procedure [10,11]. We should therefore examine the information provided by children as to their actual participation in the decision-making process. One to four months after the genetic research, just 54% of children claimed that their parents had asked their opinion on participation in the studies. Some 42% stated that “nobody asked my opinion about participating in the study”. However, 39% thought that “parents and children should both be asked for permission”, and 33% that “children my age should always give permission”.

### What can be learned from the Gabriel case?

As the Polish part of the GABRIEL case, followed by the study by van der Pal et al., gives limited data, we would like to avoid overgeneralisation regarding the actual process of obtaining assent in paediatric research. However, this case indicates a problem which should become the subject of further investigation. It also allows one to propose some hypotheses for further studies. The lack of assent in the GABRIEL case might have been caused by various factors. The question is why investigators did not design the study to meet international ethical requirements. Most international guidelines, such as the Declaration of Helsinki and the Oviedo Convention, are well known in Poland. Moreover, Polish law assumes the necessity of consent being obtained from children under 16 in the case of clinical trials, yet the genetic research in question did not put this possibility to use. It is likely that the failure to instigate the procedure of assent was caused by some social and structural factors. Despite the fact that Poland is a member of the European Union and has implemented the European Commission Directive concerning Good Clinical Practice, it still faces many problems characteristic of a transition society. These aspects have been analysed elsewhere [29]. However, we suspect that this may be a more general phenomenon. If researchers’ priority is a high level of efficiency, then many procedures and ethical suggestions are considered as an additional administrative burden. When a recommendation is ambiguous and not enforced by law, a requirement unclear and vague, then it is also not obvious how it should be applied in practice. Subtle ethical distinctions can then become much less applicable in practice. Since the local legislation did not foresee the need to gain assent, and international guidelines in this respect remain vague, this genetic research also did not contain such a procedure. Our hypothesis is that clear information concerning the minimum age at which it is required to obtain assent will make it more likely that children are asked for assent. The age threshold complements the concept of personalisation and gives a clear suggestion for RECs as well as researchers. However, we realise that a fundamental difficulty here is in designating such an age. Let us therefore take a look at research assessing the capacities of children concerning their understanding of medical procedures.

### Capacities of children according to studies

Research on the capacity of children in the context of biomedical studies is varied in terms of methodology and the composition of the groups studied (Table 1). Indeed, some studies do not even distinguish between the terms “assent” and “consent”. It is therefore difficult to systematically compile and compare the results of these studies [30]. Nonetheless, the conclusions presented in all these reports give useful information about children’s capacities of participation in the decision-making process.

**Table 1:** Capacities of children according to studies

**Author**	**Year**	**Number of subjects**	**Age**	**Question posed to children (example)**	**Conclusion**
Weithorn L, Campbell SB	1982	96	9-21	“What happens if a person is taking insulin and misses one injection?”	“Children as young as 9 appear able to participate meaningfully in personal health-care decision making”.
Abramovitch R et al.	1991	163	5-21	“Should you be in a study if you didn’t like it and your mother did?”	“If the instructions are given clearly and the study is not excessively complex, most children as young as 5 are capable of understanding what they will be doing and therefore have the capacity to give their assent or dissent to the research”.
Susman EJ et al.	1992	44	7-20	“What are the side effects of taking your treatment?”	“Although we found no age differences, developmental differences do exist among children, adolescents, and adults […]. Adolescents and young adults were no better than children in comprehending abstract concepts”.
Ondrusek N et al.	1998	18	5-18	“What good things might happen to other people because you are in this study?”	“In subjects younger than 9 years of age, understanding of most aspects of the study was found to be poor to non-existent”.
Miller S	2000	6	7-12	“The children were […] asked to talk about their likes and dislikes”.	Researchers should not “underestimate the awareness and maturity that some children possess when addressing issues of concern to themselves”.
Geller G et al.	2003	37 dyads of parents and children	10-17 with parents	No information regarding questions posed.	“Most children wanted or expected some degree of parental input, but still thought the final decision should be theirs”.
Burke TM et al.	2005	251	6-13	“Can you think of any good things about being in the study?”	“By creating age appropriate modules of information, children as young as six years can understand potentially difficult and complex concepts […] associated with participation in biomedical research”.

The majority of studies assessing children’s competence in understanding information related to research focus on children, adolescents and young adults between around 7 and 20 years old. Most of these studies support the hypothesis that children are more capable than usually thought. Lois A. Weithorn and Susan B. Campbell examined the competency to make medical decisions of 96 subjects aged 9 to 21. They concluded that 9-year-old children are able to “participate meaningfully” in the healthcare decision-making process [31]. Based on the similar results of a study undertaken with six children aged 7 to 12 years old, Sue Miller advocates appropriate assessment of children’s awareness and appeals against underestimating their abilities to assent [32]. The research conducted by Gail Geller et al. also provides data supporting the statement that children as young as 10 want to participate in the consent process. This willingness is in many cases also supported by parents, who often wish to involve their children in the entire process of consent/assent [33].

Several studies have also assessed younger children’s capacity for comprehension of treatment and research. One of these questioned young children’s capacity to participate in a medical decision-making process. This was a pilot study conducted by Nancy Ondrusek et al. performed with the participation of 18 healthy children, the focus of which was on an assent process for non-therapeutic medical research [34]. According to the authors of this study, most children younger than 9 years old are not able to participate in the decision-making process in a substantial way. Other research offers a positive assessment of young children’s capacity for understanding the essence of medical procedures and associated important information.

Rona Abramovitch et al. examined the children’s decision-making process regarding participation in psychological research in a series of studies with 163 people aged 5–12 [35]. In one of these studies the subjects included 21 children of 5 and 6 years old. Some 62% of these children gave adequate answers to the researcher’s questions, and most of them gave a reasonable account of what would happen in the study after it had been described to them by researchers (but before the children had participated in it). Only 10% of 5–6 year-olds were able to understand why the study was being conducted. The authors concluded that in the case of clear instructions most children aged 5 have the capacity to give assent or dissent to participation in psychological or medical research. Tara M. Burke et al. evaluated understanding of the risk and benefits associated with biomedical research among 251 children aged 6–15 and 237 adults [36]. For many answers, researchers found significant differences between age groups. The youngest group consisted of children aged 6–9 (“the youngest”). Only 24% of them defined the term “research” appropriately (72% of adults were able to define it), while 73% understood the purpose of the study. Some 62% of them were able to provide a reasonable response to the question “What will the doctors and nurses do if I decide to be in the study?”, while 56% were able to reasonably describe the benefits (“any good things about being in the study”) and 64% the risks (“any bad things about being in the study”) of the study. The authors concluded that the results supported their hypothesis regarding understanding complex concepts such as the risk and benefits of biomedical research among children aged 6–9.

Fixing the threshold does not imply that content of information provided while seeking assent should not be influenced by considerations of development. For example, Elisabeth J. Susman et al. examined competences for reasonable assent and consent for participation in biomedical research among 44 children and young adults (aged 7–20) [37]. The authors concluded that the study subjects more clearly understood concrete information and were less knowledgeable about elements of informed consent that assessed abstract information. It would be natural to use this kind of information in selecting optimal information to be communicated during the assent procedure.

In addition to differences in terms of methodology, there are also other factors which make a clear comparison between all the cited studies difficult. For instance, the standardised tests set by developmental psychologists may not allow children to understand some questions, while more open questions followed by discussion with young participants may more fully explore children’s capacities. Some studies implied a lack of understanding among children based on their incapacity. Nevertheless, the problem may lie in inadequate explanations. Another important factor is the difference between the capacities of healthy and young children with long-term illnesses. This may help to explain the results of Ondrusek et al.’s pilot study based on research with healthy children.

### Personalised assent: a proposed modification

The studies on children’s capacity to understand information concerning research carried out to date have not given clear pointers regarding the age from which assent must absolutely be obtained in paediatric research. Nonetheless, most of them point to the rather considerable capacities of 5–7 year-old children. Based on this limited data we could propose an age threshold: children aged 5–7 should be asked to assent to their participation in biomedical research. We call this threshold a school-age threshold. We suggest that in some situations, where necessary, RECs/IRBs could reduce the suggested threshold. Should new data be obtained regarding capacities of children younger than 5 years old, this suggested threshold could also be reduced.

Commentators favouring a more individualised approach might object to our proposal and argue that the idea of personalised assent fulfils the recommendation concerning each child being treated in a way that is adapted to individual expectations and level of development. The example of the part of the GABRIEL project research carried out in Poland, however, illustrates that suggestions regarding children’s participation in the decision process could be insufficient. The key for both RECs/IRBs and researchers is clear and unambiguous solutions. In the United States and United Kingdom, some institutions suggest that assent must be obtained from school age. Designating such a limit brings with it the threat of over- or underestimating children’s capacity to make important decisions [12,38-40]. We therefore propose connecting the suggested age threshold with personalisation by empowering RECs/IRBs and researchers to reduce that minimum age in certain situations. The complexity of the study, the child’s previous experience and similar factors impact a child’s ability to assent. RECs/IRBs could change the suggested threshold in these situations. Researchers should be encouraged to discuss the details of children’s participation with them and personalise the way in which all provided information is communicated. In our view this modification of personalisation by introducing a suggested requirement of assent starting at a certain age threshold is more adequate.

There are several arguments in favour of our proposal of modification of personalisation of assent. The most important is that of clarity. If a requirement is not enforced by law, then its impact depends greatly, among other factors, on the clarity of guidelines. An age threshold with the possibility of personalisation of assent is more likely to serve the interest of children better than completely flexible criteria of personalisation. The more vague a guideline, the more likely it will not be followed by RECs/IRBs and researchers. Moreover, future possible audits aimed at identification of assent would be based on a clear requirement.

The second argument is that of comprehensiveness of social rules. In similar situations social rules based on age thresholds do exist, and we would suggest that in paediatric research too there is no sufficient reason to object to this. We accept age limits in many other situations in society, although there is always the risk of arbitrariness. Children attend pre-school from a certain age, for example. Furthermore, many countries have an education requirement categorically connected to the age criterion, while the right to acquire a driving licence at a certain age is similarly arbitrary. The degree of maturity and responsibility of people aged 18 or 21 also varies considerably. Nevertheless, such legally designated age limits are accepted. The third argument is that of minimising subjective assessment. Deciding on a suggested age threshold once may be less subjective and much less arbitrary than if RECs/IRBs or researchers assess whether to allow an individual to join a particular procedure in each case. The final argument is that of efficiency: a clearly marked age threshold makes action and decision-making processes easier and more straightforward.

### Summary

The moral concept of personalisation of assent needs a more practical approach in ethical guidelines and recommendations. We therefore propose modifying it with additional suggestions about the school-age threshold. This straightforward recommendation makes assessment by RECs and IRBs more efficient and predictable. A suggested age threshold minimises subjective assessment regarding children’s capacity to assent and makes the concept of assent more consistent with other social rules. Most importantly, this clear recommendation is more likely to be followed, and thus to serve children’s interests better than vague and very flexible recommendations of personalisation of assent.

## Abbreviations

REC: Research ethics committee; IRB: Institutional review board.

## Competing interests

The authors declared that they have no competing interests.

## Authors’ contributions

MW coordinated the overall work of the group and wrote the first draft of the manuscript. All the authors revised the manuscript and made a substantial contribution to the intellectual content. All the authors approved the final version of manuscript.

## Pre-publication history

The pre-publication history for this paper can be accessed here:

http://www.biomedcentral.com/1472-6939/15/44/prepub
